# Finite Element Method for the Evaluation of the Human Spine: A Literature Overview

**DOI:** 10.3390/jfb12030043

**Published:** 2021-07-31

**Authors:** Symeon Naoum, Angelo V. Vasiliadis, Christos Koutserimpas, Nikolaos Mylonakis, Michail Kotsapas, Konstantinos Katakalos

**Affiliations:** 1 251 Air Force General Hospital, Department of Orthopedic Surgery, Attiki, 11525 Athens, Greece; chrisku91@hotmail.com; 2 School of Medicine, Aristotle University of Thessaloniki, 54124 Thessaloniki, Greece; vasiliadis.av@gmail.com; 3 2nd Department of Orthopaedic Surgery, General Hospital of Thessaloniki “Papageorgiou”, 56403 Thessaloniki, Greece; 4 Academic Orthopedic Department, General Hospital of Thessaloniki “Papageorgiou”, 56403 Thessaloniki, Greece; nikmpalias@hotmail.com; 5 Department of Orthopedic Surgery, General Hospital of Naousa, 59200 Emathia, Greece; mkotsapas1@gmail.com; 6 Laboratory for Strength of Materials and Structures, Department of Civil Engineering, Aristotle University of Thessaloniki, 54124 Thessaloniki, Greece; katakaloskostas@gmail.com

**Keywords:** finite element method, orthopedic applications, spine, scoliosis, osteoporosis, fracture

## Abstract

The finite element method (FEM) represents a computer simulation method, originally used in civil engineering, which dates back to the early 1940s. Applications of FEM have also been used in numerous medical areas and in orthopedic surgery. Computing technology has improved over the years and as a result, more complex problems, such as those involving the spine, can be analyzed. The spine is a complex anatomical structure that maintains the erect posture and supports considerable loads. Applications of FEM in the spine have contributed to the understanding of bone biomechanics, both in healthy and abnormal conditions, such as scoliosis, fractures (trauma), degenerative disc disease and osteoporosis. However, since FEM is only a digital simulation of the real condition, it will never exactly simulate in vivo results. In particular, when it concerns biomechanics, there are many features that are difficult to represent in a FEM. More FEM studies and spine research are required in order to examine interpersonal spine stiffness, young spine biomechanics and model accuracy. In the future, patient-specific models will be used for better patient evaluations as well as for better pre- and inter-operative planning.

## 1. Introduction

The finite element method (FEM) represents a computer simulation method developed for solving problems in civil and aeronautical engineering [1,2]. Applications of FEM have been used in numerous medical areas [3]. Nowadays, FEM has become a useful tool in orthopedic surgery, helping surgeons to better understand biomechanics in healthy and pathological conditions. It has the additional benefit of prediction of the changes in mechanical stress distribution around the implanted areas, helping prevent future pathologies due to an incorrect implant position [4].

**Figure 1:**
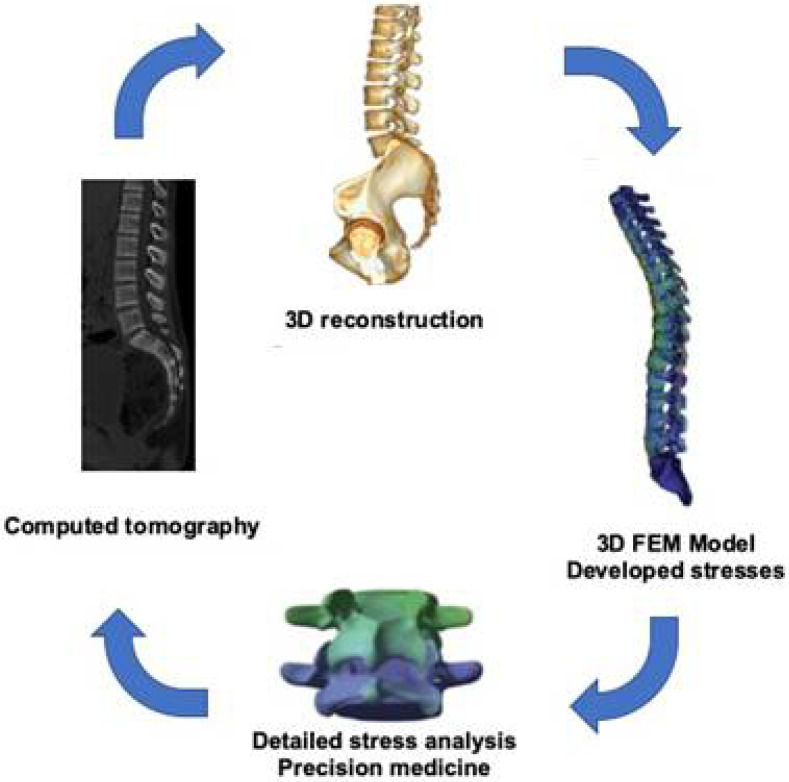
The sequence to achieve 3D lumbar spine images from the computed tomography scan and the application of the finite element method (FEM)-based stress analysis.

FEM was first applied in orthopedic biomechanics in the early 1970s to estimate stresses in the human skeleton. Additionally, during 1980–1990, FEM was used in order to investigate bone remodeling [5,6]. Computing science and technology have improved over the years, so that more complicated problems, including those of the spine, can be analyzed and studied. The first spine model was published in 1957 for pilot ejection studies [6]. Since then, numerous spring-mass models have been created, including dampers for intervertebral disc representation [4]. The first promising disc model was proposed by Orne and Liu in 1970 [3,7]. Figure 1 shows a basic diagram of digital lumbar spine reconstruction coming from computer tomography imaging. The obtained images were converted into a stereolithography (STL) file, or a 3-dimensional drawn format, from which it was then possible to complete the stress analysis by FEM (Figure 1).

**Figure 2:**
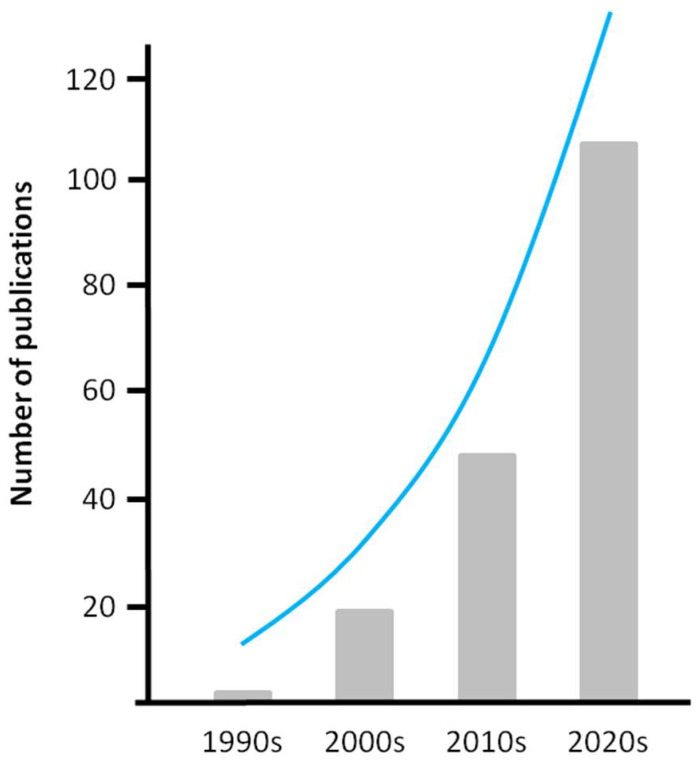
The numbers of publications from a PubMed search from late 1990s through 2020s that addressed the application of the finite element method to the evaluation of the spine.

As Figure 2 shows, the number of articles on the application of FEM to the study of the spine found in a PubMed database search using the keyword “finite element method” and “spine” has increased five-fold from the 2000s through to 2020.

The purpose of this literature overview is to emphasize FEM’s contribution to studying the biomechanics of the spine, as well as to present its various applications. We conducted a meticulous search for articles listed in the PubMed database, using mesh terms such as “finite element method or finite element analysis”, “application of FEM” and “spine”, with a cut-off date of July 2021. Citations in each article were reviewed to retrieve further references which had not been identified during the initial search. The present literature overview is limited to papers written in English and published in peer-reviewed journals. Duplicate as well as irrelevant studies (e.g., papers investigating the use of FEM in different joints) were excluded. In particular, the possibilities and applications of FEM in scoliosis, vertebral fractures, degenerative disc disease and osteoporosis are mentioned. Finally, we discuss the objective weaknesses of FEM, as well as its future development and improvement.

## 2. Functional Anatomy of the Spine

The spine is an anatomical structure with three-dimensional (3D) movements; it maintains the erect posture and supports considerable loads. It also modifies its mechanical characteristics based on the forces applied; hence, it acts as a “viscoelastic structure” [8]. The vertebral body comprises an anterior segment of cylindrical shape and a dorsal part. Cancellous bone has a highly elastic behavior for a large range of stress rates as the elastic moduli and its strength depends on its density. The width and depth of vertebral bodies are inversely proportional due to the rising axial loads [9]. The intervertebral discs consist of the nucleus pulposus centrally and the annulus fibrosus cyclically. The main role of the annulus fibrosus is structural support and consists of concentric layers of collagen fibers, helically wound. This structure results in an equal load distribution within the disc from concentric axial forces. Eccentrically placed forces provoke the displacement and bulging of the annulus on the side of the applied load, with an associated opposite displacement of the nucleus pulposus. The orientation of annular fibrosus fibers varies and differs, resulting in improved resistance to shearing and rotational loads [9].

Apart from the upper cervical spine, spinal units are connected by many ligaments with non-linear elastic responses. They are passive stabilizers, providing translational and tension-band support at the spinal column. The tension-band support is the result of the ligament’s tensile strength and it acts through the moment arm. The anterior longitudinal ligament (ALL) and posterior longitudinal ligament (PLL) are the main stabilizers of the vertebral bodies. The ALL has strong physical properties and its position provides a moment arm resisting extension. The PLL provides extension of the length of spine, but it has less strength than the ALL [9].

## 3. Pathology of the Spine

### 3.1. Scoliosis

**Figure 3:**
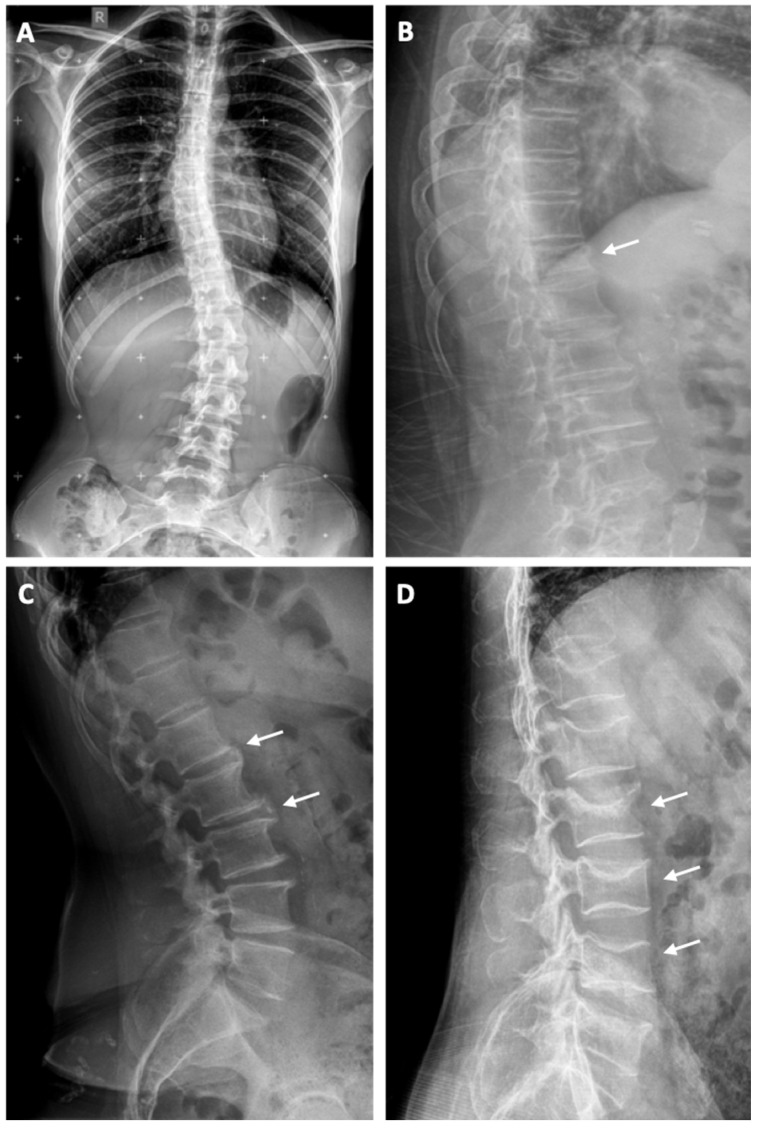
Assessment of the plain radiograph features with spine abnormalities in different patients. (**A**) Anteroposterior long-cassette radiograph of an adolescent idiopathic scoliosis in a 15-year-old patient with Risser 3; (**B**) Lateral plain lumbar spine x-ray reveals a typical wedge-shaped vertebral deformity (white arrow), indicating a L1 fracture; (**C**) Lateral lumbar spine plain x-ray reveals moderate disc space narrowing and anterior osteophytes (white arrows), indicating degeneration of the lumbar disc; (**D**) Lateral lumbar spine plain x-ray reveals biconcave deformity of their endplates and loss of more than 30% of the vertebral height (white arrows) in a patient with idiopathic osteoporosis.

Scoliosis is a pathological lateral curvature of the spine, while vertebral bodies are in rotation in the transverse plane (Figure 3). Most scoliosis cases are characterized as idiopathic, while others can be caused by neuromuscular diseases, trauma or congenital conditions [10]. Scoliosis is defined as a curvature of the spine in the coronal plane of more than 10° [11]. Severe rigid scoliosis may lead to pathological processes, such as early degeneration, injury of the vertebral column, and incomplete or even complete paralysis of the lower extremities [12].

### 3.2. Fracture

Spine trauma is an incident followed by high morbidity and mortality, as well as with additional severe consequences (Figure 3). The risk of spinal cord injury is greater in cervical spine than thoracic or lumbar spine trauma. Management of spinal cord injuries varies from external bracing with activity limitation, to more demanding approaches and interventions. The management usually differs among trauma centers, but the main goal is to select the least invasive technique for the stabilization of the injured segment [13].

### 3.3. Degenerative Disc Disease

Numerous genetic, biomechanical and anatomical variations are reported to be associated with degenerative disc disease (Figure 3). Degenerative alterations are sub-categorized as annular fissures, degeneration and herniation [14]. Degenerative cervical spine issues may be managed by dividing patient complaints into axial neck pain, myelopathy, radiculopathy or even a combination of these conditions. It is very important to understand the natural history of these disorders as well as the treatment options that can be taken into account [15].

### 3.4. Osteoporosis

Osteoporosis is defined by low bone mass and disorganization of bone architecture, with the result of compromised bone strength and increased fracture risk (Figure 3). Osteoporosis is also considered a silent disease, due to the fact that symptoms are usually absent until the first fracture takes place, which is the most serious complication of this condition. Although vertebral fractures represent the most common osteoporotic fractures, they are also the most underdiagnosed. Moreover, vertebral fractures are considered risk factors for future fracture, with a fivefold increased risk of a future vertebral fracture and a twofold to threefold increased risk of other fractures [16].

## 4. Application of FEM in the Spine

### 4.1. Scoliosis

Many types of FEM have been used in order to evaluate the results of scoliosis surgery with various implants, etiology, progression of biomechanics and bracing biomechanics [17]. Biomechanical simulation of the surgical repair of a scoliotic spine may present useful information for various techniques regarding the fixation sites and force levels [18]. FEM could also enhance the knowledge and understanding of scoliosis’s development from a mechanical standpoint [19,20].

FEM applications regarding the biomechanical analysis of scoliosis are categorized as follows: (i) studies that contribute to a better understanding of the adolescent idiopathic scoliosis etiology, (ii) studies improving brace management for moderate scoliosis cases, (iii) studies ameliorating surgical management regarding severe deformities due to scoliosis, and (iv) sensitivity analysis improving FEM’s precision.

A study in 2015 used FEM in order to examine the results of neighboring load transfer before and after the surgical fusion of lumbar scoliosis with in vivo CT scans [19]. Intradiscal pressure, ROM and facet joint forces were estimated with the application of compressive loads (extension, flexion, left lateral bending, right lateral bending, left axial rotation, right axial rotation). A large effect on kinematics and kinetics was measured at the fused level [21]. Thus, the surgeon might design the most appropriate pre-surgical strategy to anticipate mid- and long-term adverse outcomes.

In addition, FEM has been used for the investigation of spinal concave–convex biases in regard to scoliosis. FEM models evaluated the stress distribution through the vertebral growth plates, vertebral wedging, and Cobb’s angle progression. Scoliosis progression with time has also been studied and simulated with the aid of FEM analysis. Despite the fact that FEM model has not been applied in everyday practice yet, it has displayed the precise geometry as well as the material properties of the spine used, and has been able to simulate scoliosis growth and depict non-progressive scoliosis. The Cotrel–Dubousset scoliosis surgical approach was also simulated by FEM analysis. Spine geometry was derived from a 3D reconstructive model, while mechanical characteristics were personalized with lateral bending tests [22].

### 4.2. Fracture

FEM’s application in complex atlantoaxial fractures has also been investigated clinically, in order to simulate the biomechanics of this injury [23,24]. Researchers studied and evaluated the development of intracanal fracture fragments in thoracolumbar burst fractures using a 3D FEM which was considered to be suitable for dynamic analysis [25,26]. Axial loading energy may result in burst vertebral fractures leading to the inability to mechanically support the anterior, as well as the middle column; this type represents a third of thoracolumbar fractures. Today, vertebral osteoporotic fractures are increasingly frequent due to the aging population [27]. Research using FEM models has been done in order to replicate trauma-related burst fractures of both normal and osteoporotic bones [28]. The main purpose of these models was to recognize the most appropriate fixation type, as well as to identify the main differences between normal and osteoporotic bones, regarding the ROM of posterior structure and implants’ stress distribution [29]. Moreover, the models showed that osteoporotic bone decreases the structure stability and increases stress at both upper and lower vertebrae, resulting in implant failure [27].

In another study, the authors evaluated the outcomes of FEM analysis in comparison to the actual compression experiment from cadaveric thoracolumbar junctions, concluding that bone strength and fracture regions may also be projected [30].

A study published in 2020 proposed a thorough FEM of the spine to better reproduce SCI resulting from vertebral fractures [31]. In particular, FEM was used to examine the strain tolerated by the spinal cord through various parameters, as well as to analyze the potential involvement of the posterior vertebral body wall. The features of ley fracture patterns associated with SCI were recognized. These can be used for the better comprehension of injuries from the biomechanical loading of the spinal cord during trauma [31].

Consequently, FEM is suitable for the analysis of compression fractures and other conditions. FEM spinal models were built from medical imaging, while a strain analysis was conducted with compression fracture models. These results suggest that spine models derived from medical images can be used for many types of analysis [29]. For instance, FEM models have been reported to be used in order to evaluate trauma mechanisms and the effect of loading rate and ligament mechanical characteristics in lumbar spine injuries [32]. The aim was to measure the impact of sudden speed and ligament characteristics on the lumbar spine reaction during flexion shearing conditions. The findings suggest that the sudden velocity exerts an effect on the trauma mechanism as well as the final injury pattern. Moreover, further anterior displacement and increased incidence of facet fracture was reported, indicating an increased risk of instability and neurological deficit. These findings, as an additional use of FEM, might also provide useful information regarding the alarming effects deriving from trauma [32].

In one recent study, whole spine FEM models, including the rib cage, were created, and a strain analysis was also conducted through compression fracture models [33]. The findings indicated that the rib cage inclusion strengthened the thoracic spine stability and that the thoracolumbar junction was more vulnerable to fractures. Thus, when spinal disorders and internal fixation are simulated in the future, analysis using spine models including the rib cage should be considered [34].

Specimen-specific FEMs, extracted from quantitative computed tomography (QCT), also have the potential to precisely estimate failure loads in the vertebra. Furthermore, the use of extended finite element modeling (X-FEM) gives the opportunity for a detailed analysis of crack outset and diffusion in numerous materials. QCT-based finite element models (QCT/FEM) can visualize vertebral architecture and geometry, as well as BMD distribution [34]. X-FEM may contribute to crack initiation analysis, without requiring repetitive adaptive remeshing or modeling of the discontinuity during crack diffusion. As a result, the QCT/X-FEM model could be adjusted to other loading conditions and could be a useful tool for future applications in fracture risk prediction in the elderly [34]. In addition, FEMs could contribute to the comparison of biomechanical features of fixation techniques, redistributed ROMs, von Mises stress of instrumentations, and intradiscal pressures (IDPs) of the nearby fragment under displacement loading [35]. FEM was created to investigate the mechanism of burst fractures under vertical impacting forces. The results provided information about burst fractures, including the initiation, propagation and termination, as well as the varieties of IDP, stress, contact force and vertebral bodies before and after burst fractures [36].

### 4.3. Degenerative Disc Disease

FEM can be used to examine the following issues relating to degenerative disc disease: (a) the etiology of intervertebral disc degeneration (IVDD), including biological and biomechanical pathogenesis, (b) the biology, biochemistry and biomechanics of IVDD, and (c) the examination of biological and surgical management for IVDD [37,38,39,40,41,42,43,44]. In particular, in a recent FEM study that ascertained the biomechanical consequences of a degenerated L4–L5 segment, it was found that abnormal loading and motion in the degenerated models enhanced degeneration in the neighboring normal segments [45,46]. Moreover, it was reported that facet joint forces in neighboring healthy segments intensified as the degree of disc degeneration rose. As a result, it can be concluded that a precise model of degenerated facet joints is essential for predicting future changes in facet joint loads following disc degeneration.

FEM models have also been performed for the biomechanical investigation of various methods for managing multilevel myelopathy of the cervical spine [47,48,49,50,51]. For instance, in a recent study, an intact C2–C7 spine model was generated. Four extra representations were evolved from the fusion model. The biomechanical characteristics of the plate and the disc of neighboring levels (C2/3, C6/7) were compared and analyzed. The study introduced biomechanical evidence about the surgical treatment of cervical myelopathy and also presented approaches for preventing or minimizing related complications [48].

In another study, FEM was used in order to analyze the biomechanical features of non-continuous Anterior Cervical Discectomy and Fusion-ACDF with non-continuous Cervical Disc Arthroplasty-CDA, comparing these two treatment options [38]. FEM models showed that vibration loading noticeably augments stresses and strains in intervertebral discs of the human spine when compared to equivalent static loading, which suggests that whole-body vibration (WBV) sets the lumbar spine at a greater risk of disc degeneration [39].

Another important contribution of FEM is the biomechanically validated prediction for both healthy and myelopathic spinal cord displacement when compared to in vivo motions [49,52]. Spinal cord strain was raised during extension in the cervical myelopathy FEM. All surgical methods were reported to affect spinal cord stress and strain [49]. Although surgery for cervical myelopathy is needed, findings show that it may not offer optimal spinal cord mechanics. This emphasizes the need for future research to evaluate the progression of post-surgery spinal cord strain and the necessity for an evolution in medical device technology for the management of cervical myelopathy [49].

The outcomes extracted from FEM are quite reliable, indicating that it can simulate, to some extent, the molecular, pathological and biomechanical characteristics of degenerated intervertrebral discs. In regard to traditional research, the calculated results of FEM are more quantitative and visualized. Additionally, FEM is typically performed to examine phenomena that can only be explained by traditional technological methods and it can reproduce the results over different time spans. Furthermore, due to its non-invasive and repeatability characteristics, FEM is considered to be superior, in some aspects, in comparison with other common research methods [53,54].

### 4.4. Osteoporosis

Vertebral body, intervertebral disc, surrounding ligaments and muscles may be simulated by FEM models and may also be used to describe spine-related biomechanical particularities, as well as to analyze the stress allocation of the vertebral sections. As far as osteoporosis is concerned, there are numerous studies that have used FEM models to assess fracture risk, treatment comparison and biomechanics in the osteoporotic bones [54,55,56,57,58,59,60,61,62,63,64]. In particular, it is reported that nonlinear CT/FEA had better distinctive ability for vertebral fractures than lumbar spine BMD by DXA and QCT [55,59]. As a consequence, CT/FEA may be useful as a substitute for DXA and QCT in depicting osteoporosis-related fractures [55,59]. On top of that, as far as osteoporosis screening is concerned, there were encouraging findings from another recent study which investigated the feasibility of using routine clinical multidetector computed tomography (MDCT) scans for administering an FEA for predicting vertebrae solidity [60,65,66].

As for osteoporosis management, CT/FEA was helpful for investigating the effects of teriparatide and alendronate medication on the lumbar spine [55,59,67]. Furthermore, a nonlinear CT/FEA study showed that vertebral compressive force by CT/FEA represented a considerably better predictor for vertebral fracture than BMD, and could be used to evaluate the effects of medication significantly earlier than BMD [55,59].

3D FEM models based on CT images have been constructed and have proved that the technique of tri-cortical pedicle screw-TCPS can be implemented in the osteoporotic thoracic vertebral body to reinforce the griping strength of screws and decrease the risk of pedicle screw loosening [56,68]. A 3D FEM of a thoracolumbar spine with an osteoporotic vertebral compression fracture (OVCF) was applied in order to analyze its biomechanical alterations with accessible and reliable stress analysis results [57,69].

Additionally, in a study published in 2019, the impact of osteoporosis on internal fixation following spinal osteotomy was examined [58]. An FEM model of the spine’s internal fixation after osteotomy was generated through CT images. Material characteristics were attributed to both osteoporosis and normal bone groups, and the loads, including axial compression, flexion, extension and lateral bending, reproduced the various conditions. In the osteoporosis model, the stress levels of vertebrae were decreased, while the stress levels of the screw/rod system were increased. The results indicated that the risk of fracture and internal fixation failure could be higher in osteoporotic spines [58].

## 5. Future Development

Due to the fact that FEM is only a digital simulation of the real conditions, it will never exactly simulate in vivo results [70]. Concerning biomechanics, there are many features that are difficult to represent via FEM [65]. Due to the wide variations in interpersonal spine stiffness, future studies should include a stiffness factor in the FEM model, aiming to better replicate patient-specific spine behavior [71]. Moreover, postural control remains an important challenge. Hence, active simulation of muscles in FEM models would upgrade the representation of spinal curve correction [22,72]. Model accuracy needs to be further improved with more clinical data. A sizeable experimental and clinical database of surgical and non-surgical patients with a variety of deformities, parameters and demographic data should be selected for FEM’s validation. In addition, models need to be generated by biomechanical data based on the age of each patient. All FEM models, up to the present, have performed adult-extracted data on spine biomechanics and material characteristics. There is inadequate data regarding young spine biomechanics due to the complexity of acquiring cadaveric specimens [22,73]. Additionally, new material properties should also be investigated in order to acquire precise dynamic spine responses, so that they can be used in future applications of FEM [74]. There is a need to standardize continuous QCT and FEA methodology improvement, and to integrate the proposed interventional thresholds with further studies [75]. The accuracy of FEA must be better established. Exposure to radiation should be further reduced by the evolution of the hardware component and by the development of new dose protocols, so that QCT and other CT-based analysis methods can gain greater acceptability [56].

With the evolution of software, complex structures can be better analyzed in the various FEM models and, simultaneously, probabilistic studies can be implemented in order to examine the response of a structural model to the lack of certainty of a specific input variable [76,77,78]. Last but not least, future FEM studies should include both descriptions of the validation process, and verification and sensitivity studies [22].

## 6. Conclusions

FEM has become a powerful tool in the field of orthopedics, providing surgeons with better knowledge and understanding of bone biomechanics, in both healthy and abnormal conditions. In particular, there is no doubt that FEM can make an important contribution to orthopedic and spine research, and it represents a highly promising method for future applications.

A series of different FEM models have been created and numerous applications have been mentioned in terms of scoliosis, vertebral fractures, degenerative disc disease and osteoporosis. However, FEM will never exactly replicate in vivo outcomes, as there are various factors that are difficult, if not impossible, to represent in a model. In the future, patient-specific models could be used for patient evaluation as well as for pre- and inter-operative planning. Computers and software with greater potential will allow more automated FEM generation from CT and MRI data, using feature extraction procedures to derive the crucial details.

## Data Availability

The data presented in this study are available on request from the corresponding author.
